# Dual-Wavelength Fluorescence Monitoring of Photodynamic Therapy: From Analytical Models to Clinical Studies

**DOI:** 10.3390/cancers13225807

**Published:** 2021-11-19

**Authors:** Mikhail Kirillin, Aleksandr Khilov, Daria Kurakina, Anna Orlova, Valeriya Perekatova, Veronika Shishkova, Alfia Malygina, Anna Mironycheva, Irena Shlivko, Sergey Gamayunov, Ilya Turchin, Ekaterina Sergeeva

**Affiliations:** 1 Institute of Applied Physics RAS, 46 Ulyanov St., 603950 Nizhny Novgorod, Russia; alhil@inbox.ru (A.K.); Vekfy@inbox.ru (D.K.); ag.orlova@mail.ru (A.O.); valeriya1000@yandex.ru (V.P.); shishkova1999@yandex.ru (V.S.); mironychevann@gmail.com (A.M.); gamajnovs@mail.ru (S.G.); ilya340@mail.ru (I.T.); sea_nnov@yahoo.com (E.S.); 2 Institute of Information Technology, Mathematics and Mechanics, Lobachevsky State University of Nizhny Novgorod, 23 Gagarin Avenue, 603022 Nizhny Novgorod, Russia; 3 Center for Skin Tumor Diagnostics and Treatment, Privolzhsky Research Medical University, 10/1 Minin and Pozharsky Sq., 603005 Nizhny Novgorod, Russia; aurum_pro@mail.ru (A.M.); irshlivko@gmail.com (I.S.); 4 Nizhny Novgorod Regional Oncological Hospital, Delovaya 11/1, 603126 Nizhny Novgorod, Russia

**Keywords:** photodynamic therapy, dual-wavelength fluorescence imaging, Monte Carlo simulations, chlorin-based photosensitizers, light transport, optical phantoms, animal studies, clinical studies

## Abstract

**Simple Summary:**

Fluorescence imaging is an efficient tool in monitoring photodynamic therapy procedures allowing us to track accumulation and photobleaching of a photosensitizer (PS). Chlorin-based PSs feature high absorption in the red and blue bands of visible spectrum. Due to spectral dispersion of light penetration depth in biotissues, fluorescence signals registered upon excitation by red or blue light are formed in different measurement volumes. We present analytical and numerical models of dual-wavelength fluorescence imaging for evaluation of PS localization depth in the cases of topical administration and intravenous injection. The results of analytical and numerical simulations are in good agreement with the phantom experiments, and are translated to the in vivo imaging, which allows to interpret experimental observations in animal trials, human volunteers, and clinical studies. The proposed approach allows us to noninvasively estimate typical accumulation depths of PS localization which are consistent with the morphologically expected values.

**Abstract:**

Fluorescence imaging modalities are currently a routine tool for the assessment of marker distribution within biological tissues, including monitoring of fluorescent photosensitizers (PSs) in photodynamic therapy (PDT). Conventional fluorescence imaging techniques provide en-face two-dimensional images, while depth-resolved techniques require complicated tomographic modalities. In this paper, we report on a cost-effective approach for the estimation of fluorophore localization depth based on dual-wavelength probing. Owing to significant difference in optical properties of superficial biotissues for red and blue ranges of optical spectra, simultaneous detection of fluorescence excited at different wavelengths provides complementary information from different measurement volumes. Here, we report analytical and numerical models of the dual-wavelength fluorescence imaging of PS-containing biotissues considering topical and intravenous PS administration, and demonstrate the feasibility of this approach for evaluation of the PS localization depth based on the fluorescence signal ratio. The results of analytical and numerical simulations, as well as phantom experiments, were translated to the in vivo imaging to interpret experimental observations in animal experiments, human volunteers, and clinical studies. The proposed approach allowed us to estimate typical accumulation depths of PS localization which are consistent with the morphologically expected values for both topical PS administration and intravenous injection.

## 1. Introduction

Fluorescence imaging (FI) is a well-established biomedical visualization technique which benefits from elimination of backscattered probing radiation due to the difference in the fluorescence excitation and emission wavelengths [1,2]. FI has become a routine tool in monitoring the accumulation and biodistribution of both endogenous and exogenous fluorescent markers [3,4] including photosensitizers (PSs). PSs are a wide class of photoactivated drugs which mediate a photodynamic reaction in biotissues with the formation of singlet oxygen and other reactive oxygen species upon irradiation at a specific wavelength [5]. Fluorescence-based monitoring of PS accumulation in biotissue after administration and its photobleaching in the course of PDT procedure manifested by a decrease in the observed fluorescence signal from the treated area implement the principles of theranostics which consist in the use of the same agent for both imaging and treatment [6]. The PS fluorescence and photobleaching rate are considered as metrics that can be used to estimate the efficiency of the PDT procedure [7,8,9], as well as to predict clinical outcomes [10,11,12].

Traditional FI approaches in PDT are based on the wide-field camera registration of epifluorescence distribution at the tissue surface under broad-beam excitation, which restricts the possibility to estimate the PS localization depth. Full three-dimensional mapping of fluorophore distribution can be achieved by diffuse fluorescence tomography [13] based on the principles of fluorescence registration for a set of different source and detector positions, and the further reconstruction of a 3D map. However, this imaging modality is efficient primarily for trans-illumination configurations of source and detector. Thus, it is mainly used in small animal studies [14], while clinical applications are significantly restricted by the low level of transmitted fluorescence. This limitation raises the need for alternative FI approaches for an in-depth PS localization independent from sophisticated imaging geometries and complicated reconstruction procedures. A convenient solution can be found in the use of dual-wavelength fluorescence excitation/emission which utilizes significant spectral dispersion of biotissue optical properties to complement information from different measurement volumes.

Most fluorescent dyes and proteins exhibit narrow excitation and emission bands, and multispectral fluorescence imaging is primarily utilized for the subtraction of autofluorescence background and spectral unmixing of different fluorophores [15,16,17]. At the same time, a fluorophore with a wideband excitation spectrum or several pronounced absorption peaks in different bands of the visible and NIR ranges has the potential to utilize optical absorption dispersion for assessment of the fluorophore localization depth. The difference in the optical properties of the biotissues in UV/blue and red/NIR spectral ranges governed primarily by blood absorption spectrum causes different attenuation of probing radiation in the case of multiple wavelength excitation.

**Figure 1:**
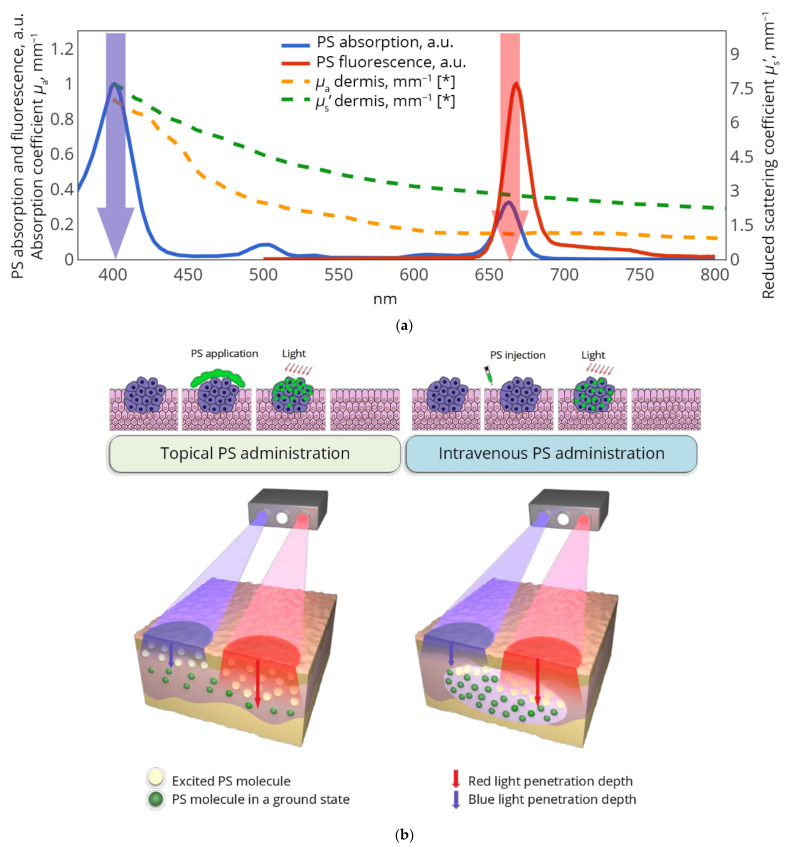
Dual-wavelength fluorescence imaging with chlorin e6 based photosensitizers. (**a**) Absorption and fluorescence spectra of chlorin e6-based PS and typical spectra of scattering and absorption coefficients of human dermis (*adopted from Salomatina et al. [23]); arrows show fluorescence excitation wavelengths employed in the proposed dual-wavelength imaging; (**b**) principles of FI with dual-wavelength excitation for topical PS administration (left) and intravenous PS injection (right).

The principle of dual-wavelength FI was initially described for fluorophores with a wide emission spectrum in a paper by Swartling et al. [18], in which the ratio of fluorescence emission at the two different wavelengths upon single-line excitation was used to estimate the embedding depth of a fluorescing sphere. Dual-wavelength excitation of fluorescence with single-wavelength detection was proposed by Miller et al. [19] and allowed for non-invasive depth estimation of a fluorescent inclusion in a mouse. This approach was further developed for monitoring the chlorin-based PSs accumulation [20,21,22]. This class of PSs features two pronounced peaks in red and blue bands of the absorption spectrum, allowing for the efficient dual-wavelength excitation of fluorescence emitted in far-red range. Figure 1a demonstrates typical chlorin e6-based PS absorption spectrum revealing absorption peaks at 402 and 662 nm (in Soret and Q-band, respectively), together with typical scattering and absorption spectra of human skin dermis [23]. One can see that human dermis absorption features around a fourfold difference at these two wavelengths resulting in a significant difference in the red- and blue-light penetration depths, and consequently, the difference in the measurement volumes: blue-light excited fluorescence originates primarily from superficial tissue layers, while red-light excited fluorescence can be registered from a larger range of depths. The ratio of these fluorescence signals has the potential to serve as a measure of the PS localization depth.

In our previous studies, we have demonstrated the perspectives of the dual-wavelength-excited fluorescence approach by numerical simulations and theoretical analysis, and have revealed the difference in the fluorescence response ratio in the cases of topical PS application and intravenous injection in in vivo experiments in mice bearing subcutaneous tumors [22,24]. It has been shown that systemic and topical methods of PS administration yield a principal difference in the outcome of dual-wavelength fluorescence imaging. In intravenous injection, the PS is delivered to tissues through the bloodflow resulting in primary PS localization within subsurface vessels shielded from direct irradiation by a superficial non-fluorescing tissue layer, while for topical administration the PS is initially accumulated within superficial and subsurface skin layers (Figure 1b).

In this paper we have generalized the concept of fluorescence imaging based on dual-wavelength fluorophore excitation applied to monitor PDT with chlorin e6-based photosensitizers. The study includes the extension of a previously developed analytical model of fluorescence signal formation [22] to account for a more realistic distribution of PS within biotissues, as well as its verification in numerical and phantom experiments, the analysis of PS monitoring in animal studies, observations from human volunteers, and a retrospective clinical study including both non-tumor and tumor pathologies. All of the PSs considered in this study are based on chlorin e6, and despite a difference in the particular formulations they are equivalent from the point of dual-wavelength imaging. The developed model has been employed for direct estimation of the PS localization depth based on dual-wavelength FI data and typical values for biotissues’ optical properties.

## 2. Materials and Methods

### 2.1. Analytical Model of Dual-Wavelength Fluorescence Sensing in Biotissues with Different Distributions of Fluorophores

Theoretical evaluation of fluorescence signal from scattering and absorbing media with distributed fluorophore is arranged similarly to [22]. Biotissue is considered to be a semi-infinite volume of uniform turbid medium illuminated from the top by a plane-wave excitation source with the intensity *I*_ex_ at the wavelength *λ*_ex_. It contains a fluorophore which distribution is characterized by a transversely uniform dependence of absorption coefficient *µ*_a,PS_(*z*, *λ*_ex_) along the longitudinal axis *z*. In the considered spectral range of 400–800 nm, optical properties of the base biotissue are characterized by spectral dependences of absorption coefficient *µ*_a_(*λ*), scattering coefficient *µ*_s_(*λ*), anisotropy factor *g*(*λ*) and refractive index *n* which is assumed to be constant in the abovementioned wavelength range. Supplementary parameters describing diffusive scattering in biotissue are the reduced scattering coefficient ${\mu^{\prime }}_{s}\left(\lambda \right)=\mu_{s}\left(\lambda \right)\left(1-g\left(\lambda \right)\right)$ and transport coefficient ${\mu^{\prime }}_{t}\left(\lambda \right)=\mu_{a}\left(\lambda \right)+{\mu^{\prime }}_{s}\left(\lambda \right)$. The absorption of excitation light by the photosensitizer at depth *z* within the elementary layer of thickness *dz* results in the emission of fluorescence at the wavelength *λ*_em_ with the efficiency characterized by the fluorescence quantum yield *ϕ*. Propagation of both excitation and fluorescence radiation in the biotissue is governed by their scattering and absorption, and can be described in the framework of the radiative transfer theory (RTT). Assuming a unidirectional incidence of excitation light and an isotropic angular emission of fluorescence, the propagation of excitation light was derived in accordance with the semi-empirical model developed by Jacques [25], while the fluorescence signal was derived within the frames of the diffusion approximation of RTT with the account of the refractive index mismatch at the biotissue–air boundary [22,26,27]. As the result, the outgoing flux of fluorescence radiation *F* at the biotissue boundary *z* = 0 normal to its surface can be derived from the expression [22]:

(1) $$F\left(\lambda_{ex},\lambda_{em}\right)=\phi I_{ex}k_{ex}\frac{sinh\left(q_{em}\right)e^{-q_{em}}}{q_{em}}\int_{z=0}^{\infty }\mu_{a,PS}\left(z,\lambda_{ex}\right)e^{-\left(\mu_{ex}+\mu_{em}\right)z}dz,$$

where *µ_ex_* and *µ_em_* are the values of the diffusion attenuation coefficient $\mu \left(\lambda \right)=\sqrt{3\mu_{a}\left(\lambda \right)\mu \prime_{t}\left(\lambda \right)}$ at the corresponding wavelengths *λ_ex_* and *λ_em_*; $q_{em}=\frac{2\mu_{em}m}{3{\mu^{\prime }}_{t}\left(\lambda_{em}\right)}$, where *m* is the factor accounting for the total internal reflectance of diffusive fluorescence light determined by formula (2.4.1) in [26]; and *m* ≅ 2.76 for the predefined refractive index *n* = 1.37. The value $k_{ex}$ is the backscattering factor evaluated in the following form [25]:

(2) $$k_{ex}=3+5.4p-2e^{-17p},\;p=exp\left(-\frac{8}{3}\frac{\mu_{ex}}{{\mu^{\prime }}_{t}\left(\lambda_{ex}\right)}\right)$$

Formula (1) is derived under the assumption that the absorption coefficient of PS is small compared to that of biotissue in the whole range of depths and wavelengths (*µ*_a,PS_(*z*, *λ_ex_*) << *µ*_a_(*λ_ex_*)).

For chlorin-based PSs with two pronounced peaks in the absorption spectrum corresponding to the wavelengths *λ*_1_ and *λ*_2_ > *λ*_1_, we introduce the ratio *R*_λ_ of fluorescence signals excited at these wavelengths and registered by the same detector, each of them normalized by the excitation intensity at the corresponding wavelength:

(3) $$R_{\lambda }=\frac{F\left(\lambda_{2},\lambda_{em}\right)/I_{ex2}}{F\left(\lambda_{1},\lambda_{em}\right)/I_{ex1}}$$

Using Equation (1), we obtain $R_{\lambda }$ as

(4) $$R_{\lambda }=\frac{\phi_{2}k_{ex2}\int_{z=0}^{\infty }\mu_{a,PS}\left(z,\lambda_{2}\right)e^{-\left(\mu_{ex2}+\mu_{em}\right)z}dz}{\phi_{1}k_{ex1}\int_{z=0}^{\infty }\mu_{a,PS}\left(z,\lambda_{1}\right)e^{-\left(\mu_{ex1}+\mu_{em}\right)z}dz},$$

where the index (*i* = 1, 2) defines the above-introduced characteristics (ϕ, *k_ex_*, and *μ_ex_*) at the corresponding excitation wavelength (*λ*_1_, *λ*_2_).

**Figure 2:**
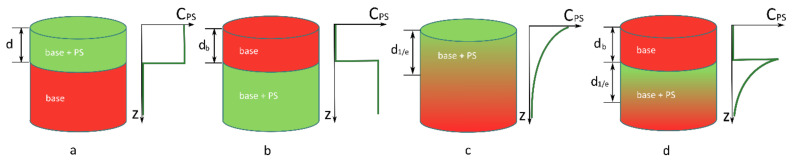
Schematics of the considered cases of PS distribution in the tissue mimicking basic examples of topical or systemic PS administration: (**a**) PS is distributed uniformly in the upper layer of biotissue of thickness d; (**b**) PS is distributed uniformly within the semispace below the biotissue layer of thickness *d*_b_; (**c**) PS concentration exponentially decreases in depth with the 1/e decay depth *d*_1/e_; and (**d**) a PS layer with an exponentially decaying concentration with the scale *d*_1/e_ is covered by the biotissue layer of thickness *d*_b_. The dependence *C*_PS_(*z*) illustrates the PS concentration in-depth profile.

In the transversely uniform problem, the ratio $R_{\lambda }$ depends on the in-depth distribution of a photosensitizer which can be quite complex. We consider four simple cases which approximately describe PS distribution mimicking basic examples of topical or systemic PS administration: (Section 2.1.1) PS is distributed uniformly in the upper layer of biotissue of thickness *d*, (Section 2.1.2) PS is distributed uniformly within the semispace below the biotissue layer of thickness *d_b_*, (Section 2.1.3) PS concentration exponentially decreases in-depth with the 1/e decay depth *d*_1/e_, and (Section 2.1.4) a PS layer with an exponentially decaying concentration with the scale *d*_1/e_ is covered by a biotissue layer of thickness *d_b_*. Schematics of all of the considered geometries together with the corresponding in-depth PS distributions are shown in Figure 2.

#### 2.1.1. PS Is Distributed Uniformly in the Upper Layer of a Biotissue of Thickness *d*

Let the PS distribution be described by a boxcar function with the width *d* and the amplitude $\mu_{a,PS,i}$, corresponding to the excitation wavelength *λ*_i_ (*i* = 1, 2):

(5) $$\mu_{a,PS,top}\left(z,\lambda_{i}\right)=\mu_{a,PS,i}\left(H\left(z\right)-H\left(z-d\right)\right),$$

where *H*(*z*) is the step function. This profile roughly describes PS accumulation in the upper skin layer, with a constant concentration within the layer *d* after topical application. In this case the ratio $R_{\lambda ,top}$ is the saturating function of the layer thickness:

(6) $$R_{\lambda ,top}\left(d\right)=\frac{\phi_{2}\mu_{a,PS,2}}{\phi_{1}\mu_{a,PS,1}}\frac{k_{ex2}}{k_{ex1}}\frac{\mu_{ex1}+\mu_{em}}{\mu_{ex2}+\mu_{em}}\frac{1-exp\left[-\left(\mu_{ex2}+\mu_{em}\right)d\right]}{1-exp\left[-\left(\mu_{ex1}+\mu_{em}\right)d\right]},$$

Detailed analysis of the $R_{\lambda ,top}\left(d\right)$ dependence is given in [22]. In particular, at small thicknesses (*d* < 1/($\mu_{ex1}+\mu_{em}$)), $R_{\lambda ,top}\left(d\right)$ does not depend on the characteristics of biotissue or the PS at the emission wavelength, and grows linearly with *d* with the slope $\frac{\mu_{ex1}-\mu_{ex2}}{2}$, which is positive due to the condition *μ*_ex_(*λ*_2_) < *μ*_ex_(*λ*_1_) when *λ*_2_ > *λ*_1_. At thicknesses exceeding z* = 3/($\mu_{ex2}+\mu_{em}$), the ratio becomes insensitive to the further increase of *d*, and saturates at the value

(7) $$R_{\lambda ,top}\left(d\to \infty \right)=R_{\lambda ,top}^{\left(\infty \right)}=\frac{\phi_{2}\mu_{a,PS,2}}{\phi_{1}\mu_{a,PS,1}}\frac{k_{ex2}}{k_{ex1}}\frac{\mu_{ex1}+\mu_{em}}{\mu_{ex2}+\mu_{em}},$$

Note that $R_{\lambda ,top}$ does not depend on the PS concentration, but on the ratio of the peak values in its absorption spectrum.

#### 2.1.2. PS Is Distributed Uniformly within the Semispace below the Biotissue Layer of Thickness *d_b_*

In this case, the PS distribution is described by the step function:

(8) $$\mu_{a,PS,bottom}\left(z,\lambda_{i}\right)=\mu_{a,PS,i}H\left(z-d_{b}\right),$$

which roughly describes subcutaneous PS accumulation after intravenous injection. The ratio (4) in this case is the exponentially growing function of the upper layer thickness *d_b_*, and it is related to the asymptotic value of $R_{\lambda ,top}^{\left(\infty \right)}$:

(9) $$R_{\lambda ,bottom}\left(d_{b}\right)=\frac{\phi_{2}\mu_{a,PS,2}}{\phi_{1}\mu_{a,PS,1}}\frac{k_{ex2}}{k_{ex1}}\frac{\mu_{ex1}+\mu_{em}}{\mu_{ex2}+\mu_{em}}e^{\left(\mu_{ex1}-\mu_{ex2}\right)d_{b}}=R_{\lambda ,top}^{\left(\infty \right)}e^{\left(\mu_{ex1}-\mu_{ex2}\right)d_{b}}.$$

#### 2.1.3. PS Concentration Exponentially Decreases In-depth with the 1/e Decay Scale *d*_1/e_

The exponential profile roughly describes the PS penetration into skin due to diffusion after topical administration [28]. Because the absorption coefficient is proportional to the absorber concentration, we define the in-depth dependence of $\mu_{a,PS}$ by the function

(10) $$\mu_{a,PS,exp}\left(z,d_{1/e},\lambda_{i}\right)=\Sigma_{PS,i}C_{PS}\exp\left(-z/d_{1/e}\right),$$

where $\Sigma_{PS,i}$ is the absorption cross-section corresponding to the excitation wavelength *λ_i_* (*i* = 1,2), and $C_{PS}$ is the concentration of PS at the biotissue surface, which can potentially depend on the PS characteristic decay depth $d_{1/e}$ when a fixed amount of PS is re-distributed in-depth from the top. The ratio (4) for the profile (10) can be calculated by integrating in (4) over a semi-infinite depth range:

(11) $$R_{\lambda ,exp}\left(d_{1/e}\right)=\frac{\phi_{2}k_{ex2}}{\phi_{1}k_{ex1}}\frac{\Sigma_{PS,2}}{\Sigma_{PS,1}}\frac{\left(\mu_{ex1}+\mu_{em}\right)d_{1/e}+1}{\left(\mu_{ex2}+\mu_{em}\right)d_{1/e}+1}$$

Note that the $R_{\lambda ,exp}\left(d_{1/e}\right)$ does not depend on the concentration at the surface $C_{PS}$, while the ratio of the absorption cross-sections is equal to the ratio of the absorption coefficients in the case of uniform distribution: $\frac{\Sigma_{PS,2}}{\Sigma_{PS,1}}=\frac{\mu_{a,PS,2}}{\mu_{a,PS,1}}$. This allows for the direct comparison of the formulae (6) and (11). Both are saturating dependencies which have equal asymptotic values at infinitely small thicknesses:

(12) $$R_{\lambda }^{\left(0\right)}\equiv R_{\lambda ,top}\left(d⟶0\right)=R_{\lambda ,exp}\left(d_{1/e}⟶0\right)=\frac{\phi_{2}\mu_{a,PS,2}}{\phi_{1}\mu_{a,PS,1}}\frac{k_{ex2}}{k_{ex1}},$$

and at large thicknesses:

(13) $$R_{\lambda ,top}^{\left(\infty \right)}=R_{\lambda ,exp}\left(d_{1/e}⟶\infty \right)=\frac{\phi_{2}\mu_{a,PS,2}}{\phi_{1}\mu_{a,PS,1}}\frac{k_{ex2}}{k_{ex1}}\frac{\mu_{ex1}+\mu_{em}}{\mu_{ex2}+\mu_{em}}.$$

However, the linear increase of the ratio $R_{\lambda ,exp}$ at small values of *d*_1/e_ is characterized by the slope $\left(\mu_{ex1}-\mu_{ex2}\right)$ which is twice larger than that for $R_{\lambda ,top}$.

#### 2.1.4. A Layer with an Exponentially Decaying PS Concentration with the 1/e Scale $d_{1/e}$ Is Located below the PS-Free Biotissue Layer of Thickness $d_{b}$

The described distribution can roughly approximate the effect of the surface photobleaching of topically applied PS after the PDT procedure. We assume the 1/e scale of the PS concentration decay $d_{1/e}$ is preserved; however, the upper boundary of the PS is shifted at the depth *z* = $d_{b}$ due to the photochemical interaction of PS with light which is most efficient at the tissue surface. In this case, the ratio $R_{\lambda ,bottom\;exp}$ becomes the function of the two variables, and can be derived by combining the assumptions made in the cases (Section 2.1.2 and Section 2.1.3):

(14) $$R_{\lambda ,bottom\;exp}\left(d_{1/e},d_{b}\right)=\frac{\phi_{2}\mu_{a,PS,2}}{\phi_{1}\mu_{a,PS,1}}\frac{k_{ex2}}{k_{ex1}}\frac{\left(\mu_{ex1}+\mu_{em}\right)d_{1/e}+1}{\left(\mu_{ex2}+\mu_{em}\right)d_{1/e}+1}e^{\left(\mu_{ex1}-\mu_{ex2}\right)d_{b}}$$

Note that the value of $R_{\lambda ,bottom\;exp}$ differs from $R_{\lambda ,exp}\left(d_{1/e}\right)$ by the multiplicative factor $e^{\left(\mu_{ex1}-\mu_{ex2}\right)d_{b}}$ containing “the depth of bleaching” $d_{b}$. The latter can be easily estimated by the relation $R_{\lambda ,bottom\;exp}$/$R_{\lambda ,exp}$ of the ratios after and before the PDT procedure.

#### 2.1.5. Estimation of the PS Depth from the Ratio $R_{\lambda }$

The monitoring of the ratio $R_{\lambda }$ by dual-wavelength fluorescence imaging in the PDT protocol allows for the tracing of the PS localization. For the PS distributions (Section 2.1.1, Section 2.1.2, Section 2.1.3 and Section 2.1.4) considered above, the inverse functions *d*(*R*_λ_), *d_1/_*_e_(*R*_λ_), *d_b_*(*R*_λ_) can be constructed, which enables the quantitative estimation of the PS localization depth if the optical properties of the base biotissue and the PS absorption spectrum $\mu_{a,PS}\left(\lambda \right)$ are known. We point out that for case (Section 2.1.1) there is no analytical solution for the inverse function $d\left(R_{\lambda ,top}\right)$, and the problem should be solved numerically. In case (Section 2.1.2), which mimics subcutaneous PS accumulation after intravenous injection, the depth $d_{b}$ of the PS upper border can be estimated from the known ratio $R_{\lambda ,bottom}$ by following Equation (9):

(15) $$d_{b}\left(R_{\lambda ,bottom}\right)=\frac{1}{\mu_{ex1}-\mu_{ex2}}\ln\left(\frac{R_{\lambda ,bottom}}{R_{\lambda }^{\left(\infty \right)}}\right),$$

where $R_{\lambda }^{\left(\infty \right)}$ is the analytical asymptotic value of $R_{\lambda }$ for an infinitely thick PS-containing slab calculated according to (7).

In case (Section 2.1.3), one can estimate the 1/e decay depth $d_{1/e}$ of the PS concentration from the measured $R_{\lambda ,exp}$ by the analytical relation

(16) $$d_{1/e}\left(R_{\lambda ,exp}\right)=\frac{R_{\lambda ,exp}-R_{\lambda }^{\left(0\right)}}{R_{\lambda }^{\left(0\right)}\left(\mu_{ex1}+\mu_{em}\right)-R_{\lambda ,exp}\left(\mu_{ex2}+\mu_{em}\right)},$$

where $R_{\lambda }^{\left(0\right)}$ is the asymptotic value of $R_{\lambda }$ for an infinitely thin PS-containing layer calculated according to (12). For the known PS absorption spectrum $\mu_{a,PS}\left(\lambda \right),$ the magnitude of $R_{\lambda }^{\left(0\right)}$ can be estimated by the ratio of the back-reflectance signals *k_ex_*_2_/*k_ex_*_1_ registered from the base medium after illumination at $\lambda_{2}$ and $\lambda_{1}$.

The case (2.1.4) describes the effect of a PDT procedure applied to the biotissue with the initial PS distribution in the form (10) when superficial light action results in the PS photobleaching from the top. The depth of photobleaching $d_{b}$ can be estimated from the value of $R_{\lambda ,bottom,exp}$ using Equation (14), provided that the ratio $R_{\lambda ,exp}$ has been measured prior to the PDT procedure:

(17) $$d_{b}(R_{\lambda ,bottom\;exp})=\frac{1}{\mu_{ex1}-\mu_{ex2}}\ln\left(\frac{R_{\lambda ,bottom,exp}}{R_{\lambda ,exp}}\right),$$

### 2.2. Dual-Wavelength Fluorescence Imaging Setup

In the phantom and in vivo experiments, a custom-built fluorescence imaging device [20,29] was employed for the registration of the fluorescence from chlorin-based PSs with the absorption and emission spectra as in Figure 1. The setup featured two LED sources at the wavelengths of 405 ± 10 nm (blue) and 660 ± 10 nm (red) synchronized with the CCD camera. For the fluorescence registration, a 772/140 filter (Semrock, Rochester, NY, USA) was used to eliminate the contribution of red excitation radiation to the registered signal. The abovementioned wavelengths correspond to the excitation and emission lines of chlorin-based PS. The probing radiation from the red LED source passed through the 641/75 filter (Semrock, Rochester, NY, USA) before the delivery to the tissue surface. Wide-field irradiation was employed for the epifluorescence excitation. The exposure time was 0.3 s for both excitation wavelengths, and LED power was 1.2 W and 1.5 W for blue and red light, respectively, resulting in probing intensities of 1.4 and 2.2 mW/cm^2^ at the object surface, correspondingly. The illumination area with the size of 13 cm × 17 cm matched the imaging area. The dark image was captured and subtracted automatically from the fluorescence images for the elimination of ambient light noise. The fluorescence response was calculated for the wavelengths *λ_ex,_*_1_ = 405 nm and *λ_ex,_*_2_ = 660 nm as the average signal over the same region of interest (ROI) with the dark level subtracted. As it was proposed earlier [20,21,22], the ratio of fluorescence responses at these two wavelengths can serve as a measure of PS localization depth because it exhibits the proportion of signals coming from deeper and superficial tissue layers.

### 2.3. Model Experiment on Biotissue Phantoms

Structured solid agarose phantoms mimicking superficial biotissue with the distributed PS were designed as the two-layer slabs of a base tissue layer and PS-containing layers. A two-layer phantom with a PS-containing top layer and a bottom base layer imitates the case of topical PS administration when it penetrates to a particular depth in the tissue. A two-layer phantom with a top base layer and a PS-containing bottom layer imitates the case of the systemic administration of PS through an intravenous injection with its primary accumulation in blood vessels and the PS absence in the superficial tissue layers weakly supplied by blood. The schematics of the phantom types are shown in Figure 2a,b. All of the phantoms featured a cylindrical shape with the radius *R* = 30 mm.

Most biological tissues are characterized by a significant difference of absorption in the red and blue ranges of the visible spectra; therefore, the phantoms were designed accordingly. The base phantoms were made by mixing agarose and water in a mass proportion of 5:400, with the further addition of lipofundin 20% and red ink (Koh-I-Noor, Czech Budejovice, Czech Republic) with volume concentrations of 17% and 0.2%, respectively. The components were mixed at room temperature, then heated up to 42 °C, poured into the custom-designed forms for controlling the produced layer thickness, and then cooled to room temperature into a rigid gel. The layer thickness varied between 0.34 and 3 mm. In a PS-containing layer, a water-based PS gel Revixan Derma (Revixan Ltd., Moscow, Russia) containing 0.1% pure chlorin e6 [30] was added to the base phantom in a liquid state to ensure a PS concentration of *C_0_* = 0.1% vol.

**Figure 3:**
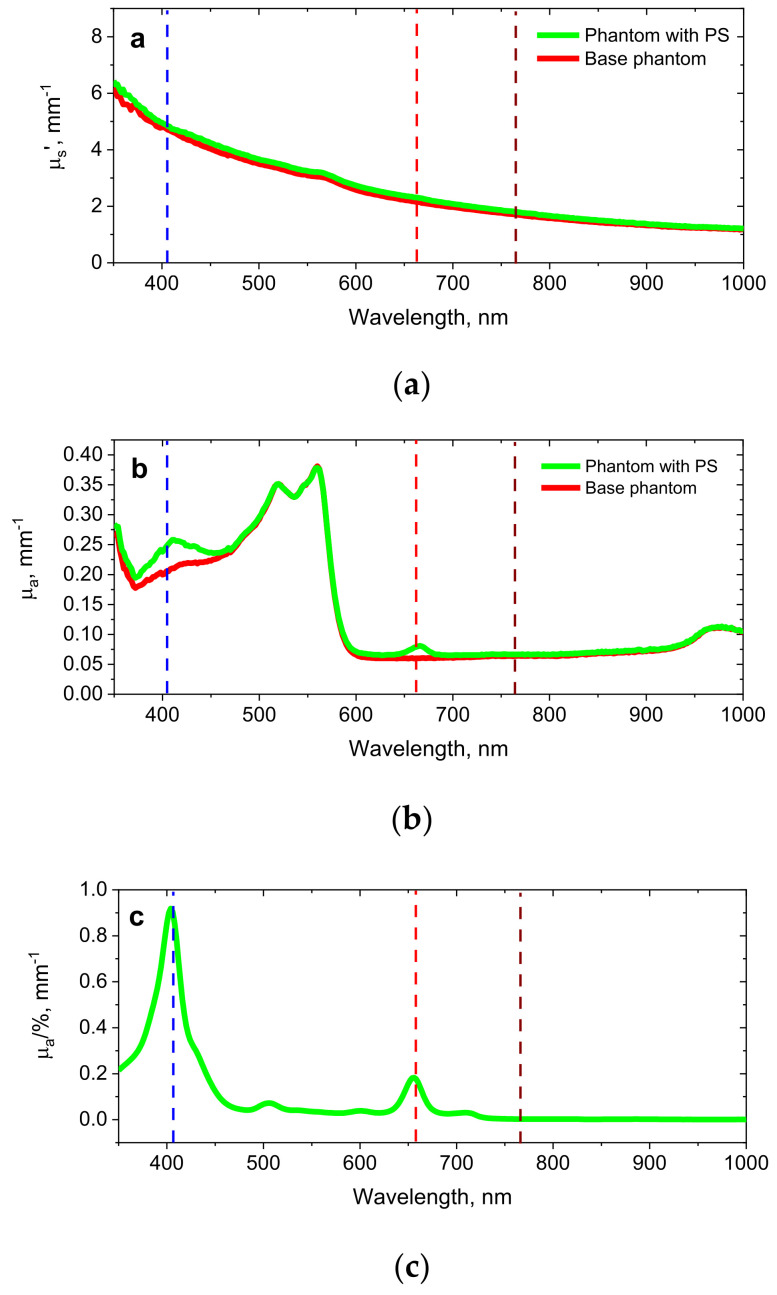
Reconstructed spectra of the reduced scattering (**a**) and absorption (**b**) coefficients of the base tissue phantom and the phantom with PS, and the absorption spectrum of the water-based photosensitizer gel Revixan Derma dissolved in purified water (**c**). Dashed lines show the fluorescence excitation (405 nm, 660 nm) and emission detection (760 nm) wavelengths.

The optical properties of the phantom layers were reconstructed from spectrophotometry measurements of the diffusion transmittance and reflectance spectra (Analytik Jena Specord 250 PLUS with an integrating sphere, Jena, Germany) from a 2 mm sample of the base medium and PS-containing medium, using a home-developed look-up table produced by massive Monte Carlo modelling. The reconstructed spectra of absorption and reduced scattering for both types of phantom layers are shown in Figure 3a,b. Additionally, the absorption spectrum of water-dissolved Revixan Derma evaluated from the measured spectrum of collimated transmittance was presented in Figure 3c. Spectrophotometry measurements demonstrate that the addition of PS to the phantom was manifested by the increased absorption in the Soret band and Q-band, while the optical properties in other spectral regions remained the same as for the base tissue. The designed phantoms feature the difference in the optical properties in blue and red bands similar to that observed in biotissues (see Figure 1a).

### 2.4. Monte Carlo Simulations

The Monte Carlo technique [31] is a common numerical simulation tool for the modelling of signals and images obtained with optical diagnostic techniques. In this study, we applied the previously developed Monte Carlo algorithm [20] for the calculation of the fluorescence response of PS distributed in biotissue. A previously proposed [20] dual-step approach to the fluorescence calculation was employed, in which, in the first step, the absorption maps of the probing radiation in tissue were calculated for both the wavelengths of 405 nm and 660 nm. The multiplication of the resulting absorption map by a factor of *μ*_a,PS_/(*μ*_a,PS_ + *μ*_a, base_) in PS-containing areas yields a map of the light dose absorbed by PS which serves as a distributed fluorescence source. In the second step, the absorption map was treated as the distributed source of fluorescence emission which was assumed to be angularly isotropic. The calculation of the fluorescence emission was performed for the optical properties corresponding to *λ_em_* = 760 nm, while the fluorescence response was calculated as the total weight of fluorescence photons per unit area exiting the tissue via the top boundary. The fluorescence signal ratio was calculated as the ratio of fluorescence responses for probing wavelengths of 660 and 405 nm. Probing irradiation with a planar wave was assumed, which corresponds to the experimental setup configuration and the analytical model. The number of probing photons launched was 10^7^.

The simulations were performed for the two-layer tissue models with the top or bottom layer containing uniformly distributed PS similar to those configurations employed in the phantom studies. In addition, a configuration with the exponential decay of the PS concentration in a uniform medium was considered. Under the condition in which the total amount of the applied PS remains constant, formula (10) was reduced to the following form:

(18) $$\mu_{a,PS,exp}\left(z,d_{1/e},\lambda_{i}\right)=\left(M_{0}\left(\lambda_{i}\right)/d_{1/e}\right)\exp\left(-z/d_{1/e}\right),$$

where *M*_0_(*λ_i_*) is proportional to the PS concentration at the tissue surface and to the PS absorption coefficient at the excitation wavelength *λ_i_*, and $d_{1/e}$ is the characteristic PS decay depth. This distribution was a closer approximation for the case of topical PS administration [28,32]. The biotissue’s optical properties were chosen in accordance with data for the human dermis [23], and are summarized in Table 1. The thickness of the top layer varied from 0.25 mm to 3 mm, while the thickness of the bottom layer was 20 mm; the transversal medium dimensions were 30 mm × 30 mm, in order to diminish edge effects. The values of the PS absorption coefficient corresponded to Revixan Derma concentration of 0.1% vol, which yields the in vivo pure drug concentration of 1 μg/g, as is consistent with the reported values [33,34].

### 2.5. Photosensitizers

The in vivo studies reported in this paper employed topical administration and intravenous injection PS, all based on chlorin e6. For the intravenous injection, a NaCl solution of Fotolon (Belmedpreparaty, Minsk, Belarus) [35,36,37,38] or an injection form of Fotoditazin (Veta-Grand Ltd., Moscow, Russia) [35,39] was used. For the topical administration, a gel form of Revixan Derma (Revixan Ltd., Moscow, Russia) [30] or Fotoditazin (Veta-Grand Ltd., Moscow, Russia) [35,39] was employed. Despite the differences in the particular formulations, all of these drugs are equivalent from the point of view of dual-wavelength imaging featuring the fluorescence spectral properties depicted in Figure 1.

### 2.6. Animal Studies

The animal study part included the analysis of fluorescence monitoring data obtained in the course of PDT procedures on the intact tissue of rabbit ears and a CT26 tumor model in laboratory mice previously reported in [24,40]. The animal studies were approved by the Ethics Committee of Privolzhsky Research Medical University (Protocol #7, 3 July 2017).

#### 2.6.1. Intact Tissue Study

The study was performed on the inner ear surface of female Russian Chinchilla rabbits (*n* = 4). Prior to the PDT procedure, the rabbits were narcotized with 0.2 mL/kg Zoletil/XylaVet injected intravenously. Each particular regime was applied in a separate rabbit ear in the three selected areas. Photosensitizer Revixan Derma was applied topically to the treated area in the amount of 0.1 mL, and was distributed evenly with a cotton swab over 2 × 3 cm^2^ of the tissue surface; 30 min after the application, the rest of the PS was removed from the tissue surface, also with a cotton swab. Prior to the PDT procedure, all of the surrounding tissues except the treated area were covered by a reflecting tape to avoid their direct irradiation. The irradiation was performed with the PDT device “Harmonia” (Laser MedCenter Ltd., Moscow, Russia), equipped with LED arrays with wavelengths of 405 and 660 nm; the fluence rate at the tissue surface was 200 mW/cm^2^ for each wavelength. The irradiation spot size was 9 mm in diameter. The accumulated light doses included in the analysis were limited by 50 J/cm^2^.

#### 2.6.2. Tumor Study

The study was performed on a tumor model of murine carcinoma CT26 in 2-month-old female Balb/c mice (*n* = 15). The cells, in the amount of 5 × 10^5^ in 100 μL PBS, were injected subcutaneously into the outer side of the left shin. The developed tumor model was subject to treatment 7 days after inoculation, when its linear size reached 3–5 mm. Prior to the PDT procedure, the animals were narcotized with the intramuscular injection of a mixture of 40 mg/kg Zoletil (Valdepharm, Val-de-Reuil, France) and 10 mg/kg XylaVet (Alpha-Vet Veterinary Ltd., Szekesfehervar, Hungary). The studied PDT regimes included doses of 250 J/cm^2^ delivered with red light (λ = 660 nm), 200 J/cm^2^ delivered with blue light (λ = 405 nm), and 250 J/cm^2^ delivered with a combination of red and blue light in equal doses (125 J/cm^2^ at 660 nm followed by 125 J/cm^2^ at 405 nm). In the protocols with intravenous injection, Fotolon PS (Belmedpreparaty, Minsk, Belarus) was administered intravenously in the amount of 5 mg/kg two hours prior to the PDT procedure [41]. In the protocols with topical application, Revixan Derma gel was applied topically in the amount of ~0.1 mL, and was distributed evenly with a cotton swab to cover all of the tumor surface (approx. 2 cm^2^); 30 min after the application, the rest of the PS was removed from the tumor surface, also with a cotton swab. Prior to the PDT procedure, all of the surrounding tissues except the treated area were covered by a reflecting tape to avoid their direct irradiation. During the application period and after the PDT procedure, the mice were kept in darkened cages. Irradiation was performed with the above-mentioned PDT device “Harmonia” (Laser MedCenter Ltd., Moscow, Russia); the fluence rate at the tissue surface was 200 and 100 mW/cm^2^ for wavelengths of 660 and 405 nm, respectively. The irradiation spot size was 9 mm in diameter.

### 2.7. In Vivo Experiment on Volunteers

The in vivo experiment was performed on the inner side of healthy volunteers’ forearms (*n* = 3; female, 24 years old; male, 27 years old; male, 38 years old; volunteers from the group of researchers). In total, 100 μL Revixan Derma gel was applied simultaneously to four neighboring areas on the surface of the skin. The area size was 2 × 1 cm^2^. Fluorescence images of these areas were obtained consequently both for the wavelengths of 405 nm and 660 nm, 200, 400, 600 and 900 s after the PS application. Prior to the fluorescence imaging, an excessive amount of PS was removed from the skin surface in order to ensure that only PS penetrated into the skin contributed to the fluorescence response. The studies were approved by the Ethics Committee of Privolzhsky Research Medical University (Protocol #13, 4 July 2019).

### 2.8. Clinical Studies

Dual-wavelength fluorescence imaging was performed in the course of PDT treatment of actinic keratosis and basal cell carcinoma performed at Privolzhsky Research Medical University in patients with indications for PDT. The clinical studies were approved by the Ethics Committee of Privolzhsky Research Medical University (Protocol #13, 4 July 2019).

#### 2.8.1. Actinic Keratosis Study

The actinic keratosis treatment protocol included topical administration of PS (Revixan Derma or Fotoditazin), followed by an accumulation period of 30 min. A total of 30 min after the administration, the PS was removed with a cotton swab, and the rest of the PS was removed with a special lotion (Revixan Ltd., Moscow, Russia). After the removal of the PS from the tissue surface, red light (λ = 660 nm) irradiation was performed with the PDT device “Harmonia” (Laser MedCenter Ltd., Moscow, Russia) described above, with a total dose varying between 50 and 150 J/cm^2^ (the intensity on the tissue surface was *I* = 200 mW/cm^2^). The total number of patients enrolled in the study was 4, with the total number of nodes amounting 14.

#### 2.8.2. Basal Cell Carcinoma Study

Basal cell carcinoma (BCC) treatment protocol included intravenous PS injection of Fotoditazin 2.5 h prior to the procedure, in the amount of 1 mg/kg. The irradiation of the tumor was performed with a custom therapeutic laser, Latus-T (Atkus, St. Petersburg, Russia), at 662 ± 1 nm. The total number of patients enrolled in the study was 5, with the total number of nodes amounting 25. The tissue surrounding the treated node was covered with an opaque material to avoid undesirable effects. The total light dose was 150 J/cm^2^, with intensity on the tissue surface of 300 mW/cm^2^.

## 3. Results

### 3.1. Comparison of the Analytical Model and Numerical Simulations

**Figure 4:**
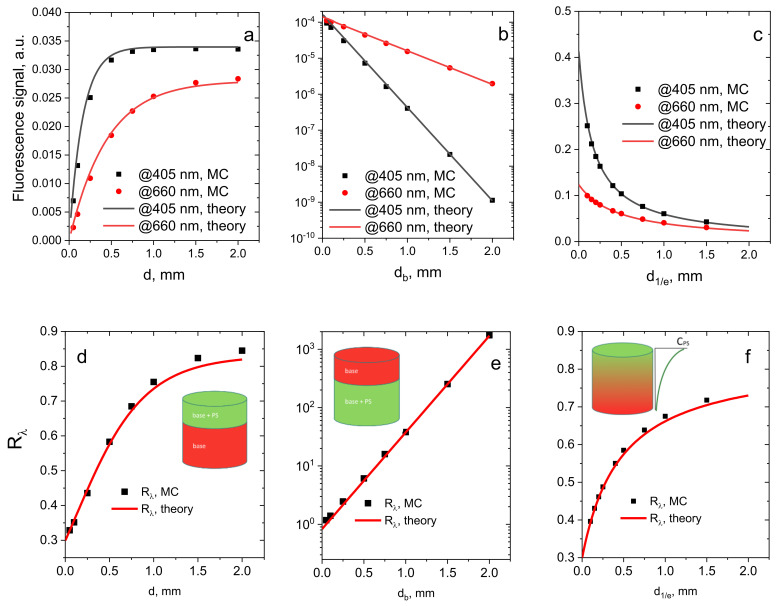
Analytical (theory) and numerically simulated (MC) dependencies of the fluorescence signals at different excitation wavelengths (**a**–**c**) and signal ratios *R*_λ_ (**d**–**f**) on the characteristic PS localization depth within human dermis mimicking biotissue, which model different methods of PS administration: (**a**,**d**) topical PS administration—uniform PS distribution within the top layer; (**b**,**e**) systemic PS injection—uniform PS distribution in the bottom layer; (**c**,**f**) topical PS administration—exponential PS concentration in-depth profile.

Numerical Monte Carlo (MC) simulations were performed in order to verify the developed analytical model of the fluorescence signals in the dual-wavelength imaging, as well as their ratios *R*_λ_ for the cases of topical and systemic PS administration. The dual-wavelength FI was modeled for the fluorescence excitation at the two wavelengths corresponding to the peaks of the Soret band (*λ*_1_ = 405 nm, “blue excitation”) and Q band (*λ*_2_ = 660 nm, “red excitation”) in the absorption spectrum of chlorin e6–based PS, with emission detection at *λ_em_* = 760 nm. Figure 4a–c demonstrate the numerically simulated and analytically calculated dependencies of the fluorescence responses at the two probing wavelengths and their ratio for the described cases (Section 2.1.1, Section 2.1.2 and Section 2.1.3), respectively, on the corresponding PS distribution parameters *d* (Figure 4a,d), *d_b_* (Figure 4b,e) and *d*_1/e_ (Figure 4c,f). Uniform (Figure 4a,d) and exponentially decaying (Figure 4c,f) distributions of PS within the top layer of biotissue imitate topical PS application, while the PS localization below the slab of non-fluorescing biotissue (Figure 4b,e) mimics systemic administration of PS by intravenous injection. For the case (Section 2.1.3), the total amount of PS is considered to be constant for different *d*_1/e_ values, such that the surface PS concentration *C*_PS_~1/*d*_1/e_. The optical properties used in analytical model and the Monte Carlo simulations are shown in Table 1, and meet the assumption of a small effect of PS on the overall medium optical properties in the analytical model. Quantum yields ϕ_1_ and ϕ_2_ were taken to be equal to 1.

For the case of uniform PS distribution within the layer of thickness *d* (Figure 4a), the fluorescence signal at both probing wavelengths demonstrates a monotonous increase followed by saturation at a large *d*, which is observed when the probing depth is smaller than the thickness of the PS-containing layer. Because the penetration depth for blue light is shorter than that for red light, the dependence of the fluorescence signal on the PS-containing layer thickness for blue excitation will saturate at smaller values of *d* compared to that for red light. Figure 4c shows similar dependencies for a more realistic case of the exponential in-depth decay of PS concentration. In this case, depth *d*_1/e_ is considered as the characteristic PS accumulation depth. Because the total amount of PS in the medium is assumed to be constant, and because it is redistributed in the tissue with the increase of *d*_1/e_, the dependencies for both blue-light and red-light excitation demonstrate a monotonous decrease.

Similarly to the fluorescence intensity dependencies on *d*, the ratio *R*_λ,*top*_ for case (Section 2.1.1) (Figure 4d) monotonously increases, reaching a constant asymptotic value. Approaching the asymptotic level means that the measurement volumes for both red and blue light are within the PS-containing layer. These results are in agreement with the previously reported studies of the top PS-containing layer [22]. Similar result is observed for the exponential PS in-depth profile, which is a more realistic model of topical PS administration (Figure 4f).

In case (Section 2.1.2), with the bottom PS-containing uniform layer which mimics the PS accumulation after intravenous injection, a monotonous decrease of the fluorescence signals versus the value of *d_b_* is observed for both wavelengths (Figure 4b) due to the decay of the amount of probing light which reaches the PS-containing region. The ratio of the fluorescence signals (Figure 4e), on the contrary, demonstrates a monotonous increase, because blue-light-excited fluorescence attenuates faster compared to red-light-excited fluorescence. As the theory predicts, this dependence has a growing exponential trend as the function of the top layer thickness with the rate determined by the difference in attenuation coefficients at two probing wavelengths (see Equation (9)). The sensitivity to the PS localization depth in this case is higher than that in the previously described case of topical PS administration. 

Figure 4a–f generally demonstrates good agreement between the theoretical and numerical dependencies, which confirms the feasibility of the developed analytical model to evaluate the PS localization depth from the measured dual-wavelength fluorescence signals. The discrepancies observed in the *R*_λ_ values in Figure 4d,f at large thicknesses of the PS layer do not exceed 3%, and are, presumably, due to the accumulated inaccuracy of the diffusion approximation taken as the basis of the theoretical model.

### 3.2. Phantom Experiments

The developed model was further verified by a phantom experiment on dual-wavelength FI with the use of solid agarose phantoms designed as described above, with spectral and fluorescent properties similar to those of biotissues. The optical properties for both the base and PS-containing layers at the wavelengths of 405 nm, 660 nm and 760 nm used in the analytical model and Monte Carlo simulations were extracted from spectrophotometry measurement (Figure 3). Typically, a direct quantitative comparison of the measured and calculated fluorescence signals is complicated by the uncertainties in the absolute values of the fluorescence quantum yields ($\phi_{1}\;\mathrm{and}\;\phi_{2}$) and PS absorption coefficients ($\mu_{a,PS,1}\;\mathrm{and}\;\mu_{a,PS,2}$), which may depend on the PS solvent [42,43]. Therefore, in order to calibrate the ratio of fluorescence signals obtained from the measurements, a reference object was prepared by casting a thin PS layer on an absorbing substrate. Being free from the depth-dependence of fluorophore localization, the *R*_λ_ value for the test object contains the information of the source intensities, quantum yields and PS absorption coefficients at two probing wavelengths:

(19) $$R_{\lambda ,ref}\approx \frac{\phi_{2}\mu_{a,PS,2}}{\phi_{1}\mu_{a,PS,1}}\frac{I_{ex2}}{I_{ex1}}$$

All of the experimentally obtained values of *R*_λ_ were normalized for the calibration coefficient $R_{\lambda ,ref}$, which allowed us to obtain the characteristic $R_{\lambda }^{c}$ independent of the parameters of the measurement setup and unknown PS properties:

(20) $$R_{\lambda }^{c}\sim \frac{R_{\lambda }}{R_{\lambda ,ref}}$$

The value of $R_{\lambda }^{c}$ is further considered in the processing of the experimental data and in the PS depth evaluation.

**Figure 5:**
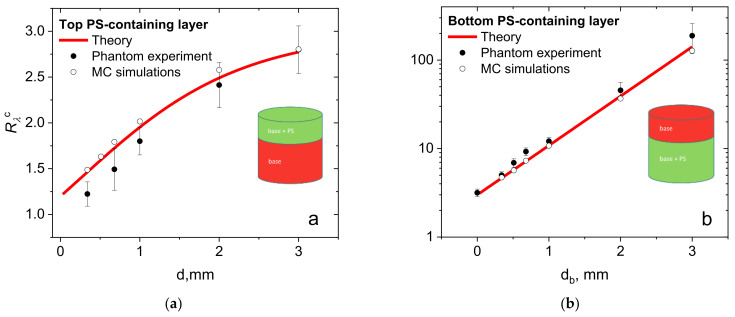
Experimentally measured fluorescence signal ratio for the agarose biotissue phantoms with (**a**) a PS-containing top layer and (**b**) a PS-containing bottom layer versus the PS localization depth, corresponding analytical dependencies and results of the Monte Carlo simulations.

Figure 5a shows the comparison of the dependencies of *R*_λ_^c^ on the thickness *d* of the phantom top layer containing PS obtained in the experiment, as simulated by MC and calculated using the developed analytical model. The MC data and analytical solution demonstrate a good quantitative agreement with the results of the phantom experiments, although discrepancy for a small thickness of the PS-containing layer may originate from inaccuracies in the optical properties reconstruction, the phantom layer thickness measurement, and the inhomogeneity of the phantom optical properties caused by its fabrication technique. Note that the absolute values of *R_λ_*^c^ in the experimental studies are different from those for *R*_λ_^c^ demonstrated for the numerical simulations (Figure 4) due to the introduction of calibration, and from the difference in the optical properties, although both dependencies on *d* have the same trend. Figure 5b demonstrates the comparison of the results of the phantom experiment with the developed analytical model and MC data for case (Section 2.1.2). The observed trend exhibits a theoretically predicted exponential dependence of the *R*_λ_ value on the thickness of the top PS-free layer (see Equation (9)).

### 3.3. Reconstruction of the Fluorophore Localization Depth from Dual-Wavelength Measurements

We performed an evaluation of the PS localization depth from numerical Monte Carlo simulations of dual-wavelength fluorescence signals, and tested the sensitivity of the reconstructed depth parameters to the variations in the biotissue optical properties employed in the reconstruction. The values of *R*_λ_ obtained from the Monte Carlo simulations were used to extract the PS localization depth using the base medium optical properties, and with the scattering and/or absorption coefficients varied by 30% in value, assuming the uncertainty in the published biotissue optical properties typically used for the interpretation of the in vivo measurements [44]. The analysis of the reconstruction accuracy was performed for the cases of the exponential in-depth decay of the PS concentration (case (Section 2.1.3) mimicking topical PS application) and a two-layer model with the bottom layer containing uniformly distributed PS (case (Section 2.1.2) mimicking intravenous PS injection) using the inverse analytical relations (16) and (15), respectively.

**Figure 6:**
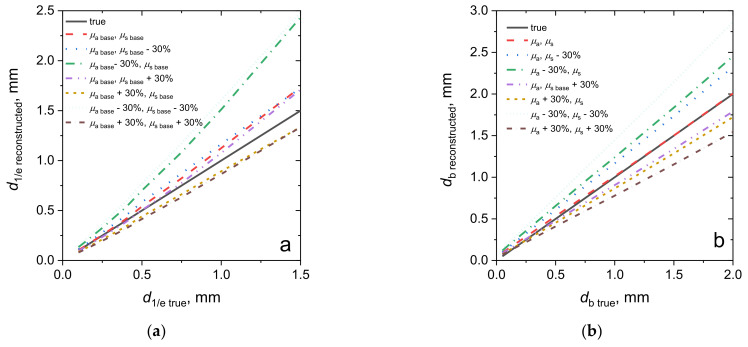
Reconstructed values of the fluorophore localization depth versus the true localization depth, depending on the medium optical properties employed for the reconstruction for cases of topical PS administration (**a**) and intravenous injection (**b**).

The results of the reconstruction of the PS localization depth *d*_1/e_ for the exponential decay of the PS concentration are shown in Figure 6a for the base optical properties employed for the reconstruction, and for cases when the scattering and/or absorption coefficients are known with an error of 30%. The inverse estimation of *d*_1/e_ by the formula (16) provides the accuracy within 10% for the characteristic depth up to 0.75 mm, given that the optical properties are exactly known. Variations in the scattering coefficient of ±30% with respect to the basic value almost do not influence the reconstruction accuracy of *d*_1/e_. On the contrary, the error in the absorption coefficient value results in a large error in the determination of the fluorophore localization, especially when *µ*_a,base_ is underestimated. Note that all of the acquired dependencies are close to linear ones.

Figure 6b shows the results of the PS localization depth reconstruction for the case of intravenous injection. For the accurately known optical properties, the inverse reconstruction (15) demonstrates the ultimate performance in the whole range of considered *d*_b_ values, while the underestimation of the optical properties’ values causes the overestimation of the localization depth, and vice versa. The simultaneous overestimation of the absorption and scattering by 30% results in about a 25% underestimation of the reconstructed value, while their simultaneous underestimation by 30% results in about a 50% overestimation of the reconstructed value.

### 3.4. In Vivo Estimations of the PS Accumulation Depth

**Figure 7:**
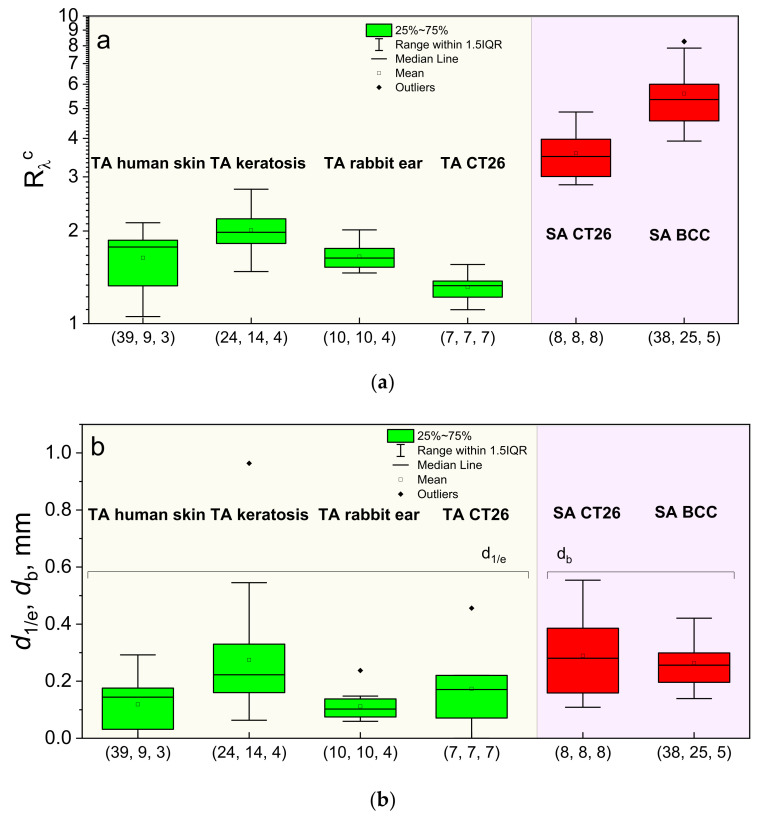
Normalized fluorescence signal ratio values of *R*_λ_^c^ detected after the PS accumulation (**a**) and the corresponding PS localization depths (**b**) for topical application (TA) and intravenous injection (SA) in laboratory animals (CT26 tumor model in Balb/c mice, inner surface of the rabbit ear), human volunteers (normal human skin) and in patients (actinic keratosis, BCC). In the brackets, total number of time points, treatment sites and independent species in the group is shown.

Figure 7a summarizes the calibrated ratio values *R*_λ_^c^ registered upon dual-wavelength fluorescence monitoring in vivo in laboratory animals, human volunteers, and patients for both cases of the topical application and the intravenous injection, while Figure 7b demonstrates the ranges of the reconstructed PS localization depths obtained from Equation (16) for the topical application and Equation (15) for the intravenous injection of PSs. The groups with the topical application of PS (human skin, rabbit ear, CT26 tumor model in mice, actinic keratosis) demonstrated typical values of *R*_λ_^c^ in the range of 1.0–3.0. Reconstruction of the typical PS localization depths for human skin using optical properties adopted from Salomatina et al. [23] resulted in the mean value of the PS penetration depth of 0.12 mm, with a maximum value of 0.29 mm, which is consistent with the typical values of human epidermis thickness varying in the range of 0.05–0.64 mm, depending on the location [45]. Note that the fluorescence measurements in the group of actinic keratosis characterized by morphological alterations in the epidermis layer and hyperkeratosis [46,47] provide higher estimations of the PS penetration depths, with the *d*_1/e_ mean value being equal to 0.27 mm and the maximum value of 0.54 mm, which is in accordance with the morphological data. Moreover, follicular extension typical for actinic keratosis [48] also stimulates deeper PS penetration. Estimations for the PS accumulation depth in CT-26 tumor-bearing mice after topical administration using the optical properties for murine tissues from Sabino et al. [49] demonstrated the mean accumulation depth of 0.17 mm, with a maximal detected value of 0.45 mm for the measured *R*_λ_^c^ values. This is in agreement with the results of our previous study [24], which demonstrated partial response of tumors to PDT treatment after topical PS administration, indicating that PS penetration through the skin to the tumor tissue occurs. Evaluation of the PS penetration depth in the rabbit ear was performed using the optical properties of human skin (because we failed to find optical properties for rabbit ear tissues), which yields the estimated penetration depth in the range of 0.06–0.24 mm, which is comparable with the human skin observations.

The estimates of the PS localization depth for intravenous injection cases for human BCC and the CT26 tumor model in mice were performed using the inverse model (15) with the optical properties for BCC [23] and mouse-skin optical properties [49]. The estimates for CT26 show an average PS embedding depth of 0.29 mm, with a maximum observed value of 0.55 mm, while for BCC the mean PS embedding depth is 0.26 mm, with the maximum being reached at 0.41 mm. Note that, in the case of intravenous injection, we evaluated the depth of the superficial layer that contains no PS, and was primarily determined by the depth of vessels delivering PS to the tumor.

### 3.5. Monitoring of PDT in Animal Studies

Because dual-wavelength fluorescence imaging can provide the estimation of PS localization, it can potentially serve as a measure of a PDT procedure’s impact depth. Owing to the pronounced absorption maxima of chlorin-based PS in the blue and red bands of the visible range, therapeutic irradiation can be performed at both wavelengths chosen for fluorescence excitation, thus providing an opportunity for controlling the impact depth. Red light PDT, which is a traditional protocol for chlorin-based PS, provides a higher impact depth (mm) however, with a lower light dose absorbed by the tissue volume compared to the blue light action. Blue light PDT can be employed for superficial action with a small impact depth but a higher absorbed light dose per tissue volume. In order to analyze the feasibility of dual-wavelength monitoring in the estimation of the PDT procedure impact depth, we evaluated the values of *R*_λ_^c^ from the fluorescence measurements prior to and after the PDT procedure delivered with either red (*λ* = 660 nm) or blue (*λ* = 405 nm) light to both normal and tumor tissue in the animal experiments.

**Figure 8:**
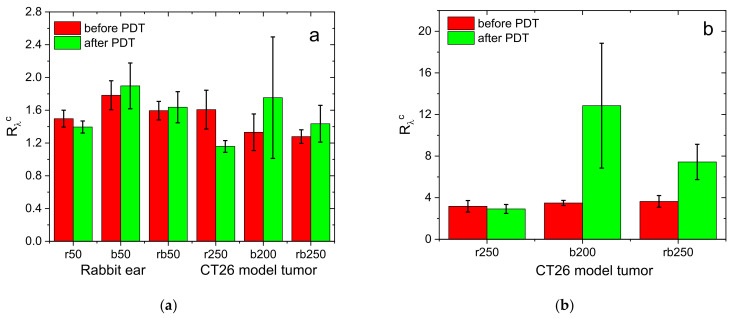
Normalized fluorescence signal ratios prior to and after PDT procedures obtained in animal studies (inner surface of rabbit ear in norm and CT26 tumor model in Balb/c mice) for topical application (**a**) and intravenous injection (**b**). The PDT regimes’ abbreviations below the bars indicate the therapeutic wavelength (‘r’ = 660 nm, ‘b’ = 405 nm, ‘rb’ = 660 nm + 405 nm) and dose in J/cm^2^.

Figure 8a shows the averaged values of *R*_λ_^c^ detected prior to and after the PDT procedures with topical PS administration observed in the rabbit ear inner surface, treated with the total dose of 50 J/cm^2^ delivered with red or blue light, or their combination in equal parts, and in the CT26 tumor model in Balb/c mice with the total dose of 200 or 250 J/cm^2^, delivered with red or blue light, or their combination in equal parts. The data for the rabbit ear do not demonstrate a significant change in the *R*_λ_^c^ value as a result of a PDT procedure. However, the red light procedure demonstrates an insignificant decrease in the *R*_λ_^c^ value opposite the blue light procedure yielding an insignificant increase in *R*_λ_^c^. The latter is presumably owing to the primarily superficial photobleaching leading to the deepening of PS-containing volume. Similar effect with a smaller magnitude is observed for the PDT protocol with the combination of red and blue light exposure.

Because the PDT dose applied to the rabbit ear was rather small, the corresponding variations in *R*_λ_^c^ as a result of the procedure were smaller than those observed in PDT of CT26 tumors where the delivered light doses were significantly higher. For the combination of two exposure wavelengths delivered in equal doses, the observed increase was smaller than that for the blue light case, owing to smaller exposure dose of the latter.

A more pronounced effect was observed in the study with tumor models in Balb/c mice with intravenous PS injection (Figure 8b) when typical antitumor light doses were delivered. Similar to the topical administration case, the red light PDT demonstrated an insignificant decrease in *R*_λ_^c^ as a result of a PDT procedure. However, the effect of the blue light PDT is manifested by the significant ratio increase owing to the much stronger sensitivity of this parameter to the PS localization depth in the case of intravenous injection (see Figure 4e and Figure 5b). This increase is governed by both the higher concentration of PS in the underlying tissue layer where major blood vessel plexuses are located, and the primary photobleaching of the PS in the superficial layers owing to the comparatively low blue light penetration depth. The blue light PDT procedure with a dose of 200 J/cm^2^ demonstrated a higher increase in *R*_λ_^c^ compared to that with red and blue light doses delivered in equal parts (125 + 125 J/cm^2^) owing to the smaller dose of blue light delivered in the latter case.

### 3.6. Clinical PDT Monitoring

**Figure 9:**
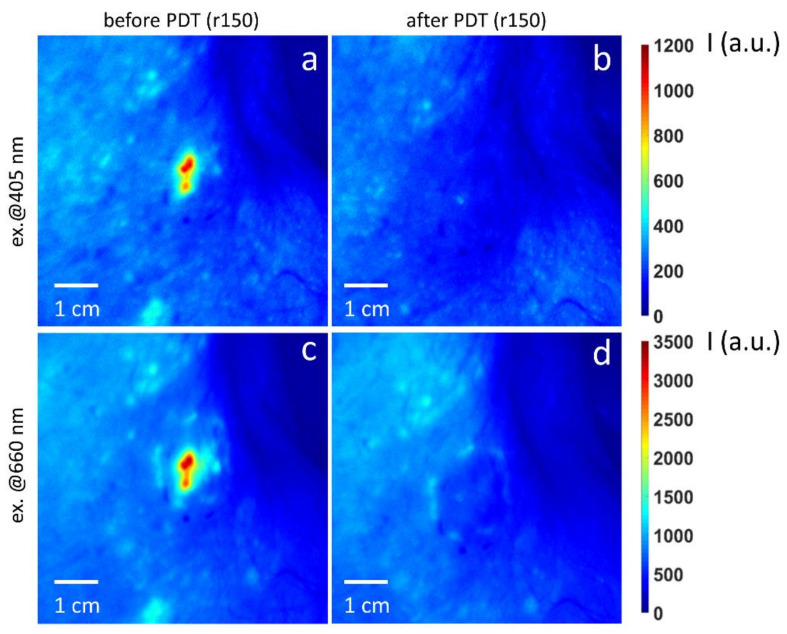
Typical fluorescence images of the BCC node site prior to (**a**,**c**) and after (**b**,**d**) the PDT procedure delivered at *λ* = 660 nm, with a dose of 150 J/cm^2^ acquired at probing wavelengths of 405 (**a**,**b**) and 660 (**c**,**d**) nm.

Clinical part of this study included the dual-wavelength monitoring of the PDT treatment of actinic keratosis and basal cell carcinoma performed with the topical application and intravenous injection of chlorin e6-based PS, respectively. The actinic keratosis treatment studies included three protocols with red and blue light irradiation, and their combination in equal light doses with the total dose of 50 J/cm^2^, while the BCC studies included a standard protocol with the total dose of 150 J/cm^2^ delivered with red light. Typical results of the dual-wavelength fluorescence monitoring of the PDT treatment of BCC are shown in Figure 9.

**Figure 10:**
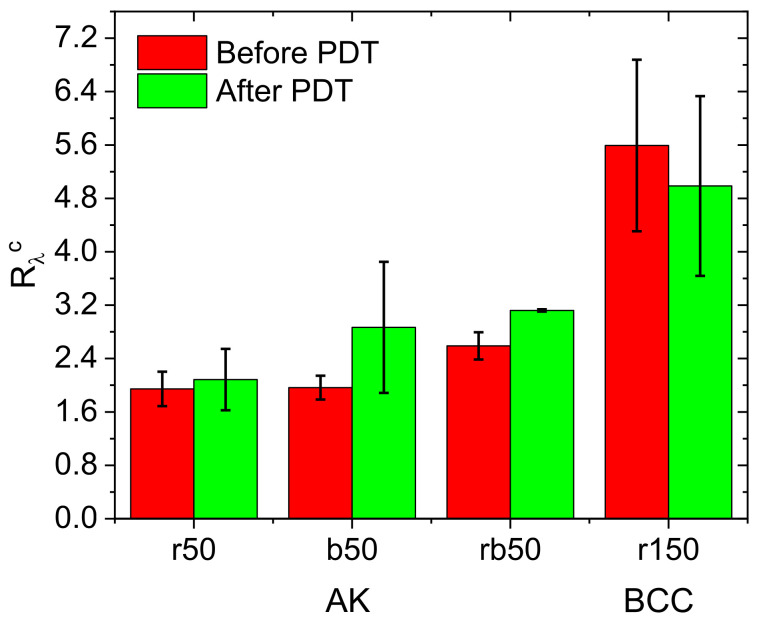
Fluorescence signal ratio prior to and after the PDT procedures obtained in the treatment of actinic keratosis (AK) with topical PS administration and basal cell carcinoma (BCC) with intravenous PS injection. The PDT regimes’ abbreviations below the bars show the therapeutic wavelength (‘r’ = 660 nm, ‘b’ = 405 nm, ‘rb’ = 660 nm + 405 nm) and dose in Joules.

High fluorescence signal in the BCC node area at both probing wavelengths demonstrates a contrast in the PS accumulation in the tumor with respect to the surrounding tissues, while low signal after the PDT procedure indicated efficient PS photobleaching. Complementary to the photobleaching observation, dual-wavelength fluorescence monitoring provides the ability for the evaluation of the PS localization depth for both of the considered clinical applications. The results of the *R*_λ_^c^ measurement prior to and after PDT both for actinic keratosis and the BCC treatment are shown in Figure 10.

For actinic keratosis, similarly to the trends previously observed in animal studies with topical PS administration, red light PDT demonstrates a weak alteration of *R*_λ_^c^ as a result of the procedure, indicating that photobleaching is quite uniform in depth because typical impact depth of the red light procedure is comparable or higher than typical depth of the PS penetration upon topical administration. The blue light procedure demonstrates a jump in *R*_λ_^c^ associated with the photobleaching of PS primarily in the superficial tissues [28,32]. The protocol involving the combination of red and blue light illumination also demonstrates an increase in *R*_λ_^c^, which is in line with the observations in animal studies with dual-wavelength protocols. The BCC treatment monitoring demonstrates an insignificant decrease of the *R*_λ_^c^ as a result of the PDT procedure. This observation is consistent with animal study observations for a red light antitumor regime with intravenous injection.

### 3.7. Estimation of the PS Localization Depth Variation as a Result of a PDT Procedure

The developed approach to the evaluation of the fluorophore localization depth for different methods of PS administration allows us to estimate the effect of a PDT procedure on the PS redistribution within tissues. As was demonstrated previously, blue light irradiation primarily causes PS photobleaching in superficial tissue layers, while red light provides a deeper impact. In order to analyze the effect of a PDT procedure with intravenous injection, the developed analytical model for a PS-containing bottom layer can be employed (Section 2, case (Section 2.1.2), Equation (9)), where the change of the reconstructed upper layer thickness is considered to characterize the change in the PS localization depth. For the analysis of the PS topical administration case characterized by the exponential decay of PS concentration, the model described in Section 2, case (Section 2.1.4) was employed, which considers PS surface photobleaching as the in-depth shift of the exponential PS profile with the preservation of the decay scale d_1/e_ (Equation (14)). As a result, for both cases, the change in the PS embedding depth can be evaluated by the corresponding *R_λ_* values before ($R_{\lambda }^{before}$) and after ($R_{\lambda }^{after}$) PDT from the relation similar to Equation (17):

(21) $$∆d_{b}=\frac{1}{\mu_{ex1}-\mu_{ex2}}\ln\left(\frac{R_{\lambda }^{after}}{R_{\lambda }^{before}}\right)$$

**Figure 11:**
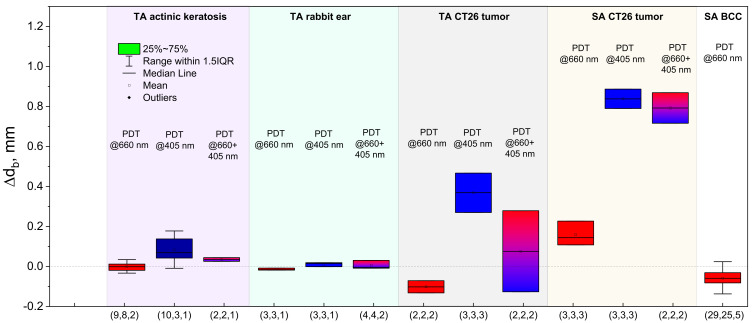
PS localization depth changes, Δ*d*_b_ measured in laboratory animals and in patients as a result of the PDT procedure (TA—topical application; SA—systemic administration). In the brackets, total number of procedures, treatment sites and independent species in the group is shown.

The results of the analysis of the localization depth changes performed for different cases are summarized in Figure 11. One can see that topical administration procedures with low doses (treatment of actinic keratosis and intact tissue of rabbit ear) provide weak variations of the PS localization depth. The highest variation is observed for the blue light treatment of actinic keratosis. The observations of the CT26 tumor treatment with topical PS administration also showed a pronounced increase in the PS localization depth for the blue light PDT regime. At the same time, the red light PDT regime shows the opposite dynamics, which indicates that the PDT-induced in-depth distribution of PS undergoes more complicated changes than the model represented by Equation (14) predicts.

The analysis of the CT26 tumor treatment in the case of intravenous PS injection shows the increase of the PS localization depth for all of the considered cases, and it is especially pronounced in the case of blue light and dual wavelength exposure, confirming that the blue light impact leads to excessive PS photobleaching at depths of up to 0.7 mm. The observation for the BCC treatment shows a general decrease in the localization depth, which could be associated with the gradual decrease in the PS concentration in deeper layers as a result of the PDT procedure or the PS diffusion.

### 3.8. Fluorescence Signal Ratio: Overview

**Figure 12:**
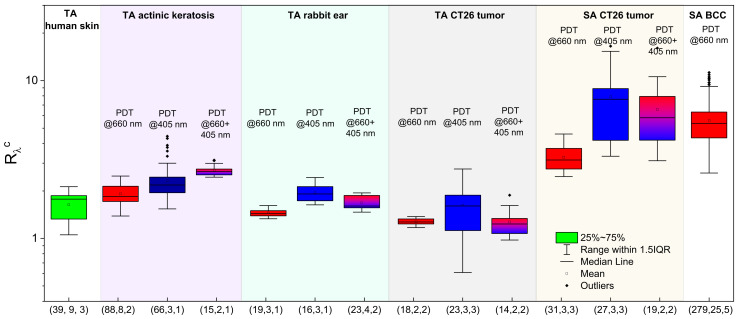
Normalized fluorescence signal ratio, *R*_λ_^c^, summarized over all of the measurements in laboratory animals, human volunteers and patients upon PS administration or/and in the course of a PDT procedure (TA—topical application; SA—systemic administration). In the brackets, total number of time points, treatment sites and independent species in the group is shown.

Figure 12 summarizes all of the normalized fluorescence signal ratio values *R*_λ_^c^ registered in vivo in laboratory animals, human volunteers and patients for the cases of topical application and intravenous injection prior to and after the PDT procedures delivered with different wavelength combinations. This figure allows us to estimate typical ranges of *R*_λ_^c^ detected in vivo. The topical application groups demonstrate typical values of *R*_λ_ in the range of 0.5–4.0, which is in line with the predictions of the analytical and numerical studies. Note that a redistribution of PS may occur as a result of a PDT procedure because the photobleaching primarily affects the superficial tissue layer, thus “shifting” the *R*_λ_^c^ values to those typical for the intravenous injection case where the superficial layer contains a small amount of fluorophore, or none. The systemic administration groups demonstrate the *R*_λ_^c^ values in the range of 3.0–20.0, which is also in line with the results of the model predictions for a PS localization depth of up to 0.5 mm.

## 4. Discussion

Fluorescence imaging, a conventional tool in biomedical research [14,50,51], is being rapidly introduced into clinical practice [12] owing to its noninvasiveness, instrumental simplicity and moderate cost. Among the clinical techniques employing FI are fluorescence image-guided surgery [52], fluorescein angiography [53] and imaging-combined photodynamic therapy (PDT) [54,55]. The limiting factors are autofluorescence and significant light scattering in biotissues, which restrict deep-tissue imaging and high-resolution localization of fluorescing areas [56]. Efforts to improve the performance of fluorescence diagnostic systems are related to the implementation of special types of fluorophores, including extremely bright quantum dots [57], fluorophores excited in NIR range [58] and nonlinear up-converting nanoparticles [59]. Another way to compete the image blurring and to improve the localization of fluorophores is to employ tomographic approaches for image formation combined with sophisticated image processing tools [60]. However, the developed techniques—such as fluorescence diffuse tomography [13,61] and multispectral fluorescence tomography [17]—are time-consuming, and are mainly applied in small animal studies due to the low signal level.

In our study, we demonstrated the feasibility of employing ratiometric dual-wavelength fluorescence imaging together with the specific type of fluorophores to quantitatively estimate the depth of the fluorescing layer located within highly scattering biotissue. The proposed ratiometric approach benefits from the significant difference in the probing light penetration depth in the blue and red spectral bands, and additionally allows us to eliminate the dependence of the analyzed signal ratio on the PS concentration within biotissue—a key parameter which is hard to measure quantitatively. We have developed simple analytical models to evaluate the fluorophore localization characteristic from the ratio of the measured epi-fluorescence signals which account for different types of in-depth fluorophore distribution mimicking realistic profiles of fluorophores’ distribution upon their accumulation in biotissues. The application of the developed models allows us to avoid complex reconstruction procedures usually employed in fluorescence tomography techniques [14,62]. The calibration procedure applied to the measured signals prior to the processing allows us to bypass the uncertainty in the ratio of the probing intensities and the fluorophore absorption coefficients at the two utilized wavelengths. The proposed approach can also be employed for the monitoring of the PS profile change as the result of photobleaching during the PDT procedure by the ratiometric measurements prior the procedure, as well as the tracking of the defined ratio in the course of the procedure. However, it is worth noting the limitations of employing the developed analytical model to accurately assess the in-depth distribution of the PS concentration. First, the model describes simple shapes of the fluorophore PS distribution (boxcar, step-like, exponential), which generally differ from real distributions in biotissues governed by different mechanisms of diffusion and accumulation in the target area, and local tissue morphological inhomogeneities, etc. In this respect, the reconstructed value of the PS localization depth serves as an effective parameter, similarly to the widely employed homogenous biotissue optical parameters such as scattering and absorption coefficients and scattering anisotropy. Secondly, the accuracy of the depth estimation strongly depends on the adequacy of the biotissue optical properties used in the model. Although the ratiometric approach and calibration procedure allow us to get rid of some parameters which are difficult to measure (such as action absorption coefficient $\phi \mu_{a,PS}$), the reconstructed depth value is quite sensitive to the uncertainty in the optical properties of the studied organ, especially the absorption coefficient, which can significantly vary even within one patient. At the same time, the data reported in the literature are typically characterized by the significant variance of biotissue absorption and scattering [23,49], which restricts the recovery of the absolute values of the PS characteristic depth; however, it provides a reliable estimation of its relative change when monitoring within the predefined location. Moreover, the combination of the reported approach with the diffuse reflectometry technique that can provide an immediate evaluation of the local tissue optical properties could significantly enhance the provided accuracy of the PS depth estimation.

We assume that the value of *R*_λ_ and its changes during the PDT procedure related to the PS redistribution in biotissue may have the potential to become one of the predictive factors of the PDT procedure outcome, together with the photobleaching efficiency [63], though this requires further studies. We also consider dual-wavelength FI as a promising diagnostic component of theranostic PDT modality which can be applied not only during treatment but also at the stage of PS accumulation after intravenous administration. Tracking of dual-wavelength fluorescence excited by low-intensity light sources during PS accumulation will provide additional information about its distribution in the area of interest: the amount of the accumulated PS is related to the intensity of individual fluorescence signals, while the change in its spatial organization influences the measured ratio *R*_λ_. Moreover, the reported approach is not limited by chlorin e6-based PSs only, but can also be applied for other types of widely employed PSs with pronounced absorption in two different bands of the visible spectrum, e.g., in 5-ALA-based [64] or benzoporphyrin derivative-mediated [65] PDT. Thus, the discussed modality of dual-wavelength FI for monitoring the fluorophore distribution in biotissues is a feasible concept of diagnostic enhancement by biophotonics tools.

## 5. Conclusions

In this paper, we present a comprehensive analysis of the dual-wavelength fluorescence imaging approach in the monitoring of PDT with chlorin e6-based photosensitizers, which gives an additional benefit of the evaluation of the fluorophore localization depth and its evolution in the course of a PDT procedure without the employment of sophisticated hardware/software tomographic modalities. The principle is based on the difference in the optical properties (primarily, absorption) of biotissue in the blue and red bands of the visible wavelength range where PS fluorescence can be excited. Owing to the different attenuation of the probing radiation at different wavelengths, the ratio of the fluorescence signals detected from the same PS allows for the estimation of its localization. We developed the analytical model of the fluorescence signal based on a semi-empirical model of light penetration combined with the diffusion approximation of the radiative transfer equation, which allowed for a simple analytical relation between the fluorescence signal ratio *R*_λ_ and the PS localization depth in several particular cases mimicking topical PS administration and intravenous injection. The model is in good agreement with the results of the Monte Carlo simulations and phantom experiments. The inverse formulae reveal the opportunity to estimate the fluorophore localization depth based on the fluorescence signal ratio, given that the optical properties of the tissue are known.

The dynamics of the *R*_λ_ value allows to noninvasively evaluate the effect of a PDT procedure that can be performed either at 660 nm or 405 nm corresponding to the absorption peaks of chlorin e6. Red light has higher penetration depth compared to blue light, thus providing deeper PDT action, while blue light primarily activates the PS accumulated in superficial tissue layers. In this connection, red light procedures feature weak variation in *R*_λ_ as a result of the irradiation, while blue light therapeutic exposure does not reach deep-seated PS which still can be sensed by fluorescence imaging, thus resulting in an increase in the *R*_λ_ value. The effect was demonstrated in laboratory animals and in patients with actinic keratosis.

Thus, the dual-wavelength approach is demonstrated to serve as an efficient tool for the monitoring of photosensitizer accumulation and photobleaching with estimations of its localization depth, and, in the case of intravenous injection, for the monitoring of the PDT effect of local microcirculation in the treated area. The reported approach was demonstrated for chlorin-based PSs; however, it has a much wider application area for different fluorophores featuring high absorption in the blue and red spectral bands.

## Figures and Tables

**Table 1:** Optical properties of biotissue layers at the fluorescence excitation and emission wavelengths employed in the Monte Carlo simulations (BT = base tissue).

λ(nm)	*μ*_a PS_, mm^−1^	*M*_0_	*μ*_a BT_, mm^−1^	*μ*_s BT_, mm^−1^	*g*_BT_	*μ*_s_‘_BT_, mm^−1^
405	0.1	0.02	0.96	38	0.8	7.6
660	0.02	0.004	0.15	14	0.8	2.8
760	0	0	0.13	12	0.8	2.4

## Data Availability

The data used in this research are available from the corresponding author upon reasonable request.

## References

[1] Andersson-Engels S., af Klinteberg C., Svanberg K., Svanberg S. (1997). In vivo fluorescence imaging for tissue diagnostics. Phys. Med. Biol..

[2] Frangioni J.V. (2003). In vivo near-infrared fluorescence imaging. Curr. Opin. Chem. Biol..

[3] Bao X., Yuan Y., Chen J., Zhang B., Li D., Zhou D., Jing P., Xu G., Wang Y., Holá K. (2018). In vivo theranostics with near-infrared-emitting carbon dots—highly efficient photothermal therapy based on passive targeting after intravenous administration. Light Sci. Appl..

[4] He S., Song J., Qu J., Cheng Z. (2018). Crucial breakthrough of second near-infrared biological window fluorophores: Design and synthesis toward multimodal imaging and theranostics. Chem. Soc. Rev..

[5] Celli J.P., Spring B.Q., Rizvi I., Evans C.L., Samkoe K.S., Verma S., Pogue B.W., Hasan T. (2010). Imaging and photodynamic therapy: Mechanisms, monitoring, and optimization. Chem. Rev..

[6] Akopov A.L., Rusanov A.A., Papayan G.V., Kazakov N.V., Gerasin A.V., Urtenova M.A. (2017). Endobronchial photodynamic therapy under fluorescence control: Photodynamic theranostics. Photodiagn. Photodyn. Ther..

[7] Weston M.A., Patterson M.S. (2013). Monitoring oxygen concentration during photodynamic therapy using prompt photosensitizer fluorescence. Phys. Med. Biol..

[8] Sheng C., Jack Hoopes P., Hasan T., Pogue B.W. (2007). Photobleaching-based dosimetry predicts deposited dose in ALA-PPIX PDT of rodent esophagus. Photochem. Photobiol..

[9] Jacques S.L., Joseph R., Gofstein G. (1993). How photobleaching affects dosimetry and fluorescence monitoring of PDT in turbid media. Optical Methods for Tumor Treatment and Detection: Mechanisms and Techniques in Photodynamic Therapy II.

[10] Warren C.B., Lohser S., Wene L.C., Pogue B.W., Bailin P., Maytin E. (2010). Noninvasive fluorescence monitoring of protoporphyrin ix production and clinical outcomes in actinic keratoses following short-contact application of 5-aminolevulinate. J. Biomed. Opt..

[11] Piffaretti F., Zellweger M., Kasraee B., Barge J., Salomon D., Van Den Bergh H., Wagnières G. (2013). Correlation between protoporphyrin IX fluorescence intensity, photobleaching, pain and clinical outcome of actinic keratosis treated by photodynamic therapy. Dermatology.

[12] Gamayunov S., Turchin I., Fiks I., Korchagina K., Kleshnin M., Shakhova N. (2016). Fluorescence imaging for photodynamic therapy of non-melanoma skin malignancies–a retrospective clinical study. Photonics Lasers Med..

[13] Corlu A., Choe R., Durduran T., Rosen M.A., Schweiger M., Arridge S.R., Schnall M.D., Yodh A.G. (2007). Three-dimensional in vivo fluorescence diffuse optical tomography of breast cancer in humans. Opt. Express.

[14] Ntziachristos V. (2006). Fluorescence molecular imaging. Annu. Rev. Biomed. Eng..

[15] van den Berg N.S., Buckle T., KleinJan G.H., van der Poel H.G., van Leeuwen F.W. (2017). Multispectral fluorescence imaging during robot-assisted laparoscopic sentinel node biopsy: A first step towards a fluorescence-based anatomic roadmap. Eur. Urol..

[16] Pu H., He W., Zhang G., Zhang B., Liu F., Zhang Y., Luo J., Bai J. (2013). Separating structures of different fluorophore concentrations by principal component analysis on multispectral excitation-resolved fluorescence tomography images. Biomed. Opt. Express.

[17] Kleshnin M., Turchin I. (2013). Fluorescence diffuse tomography technique with autofluorescence removal based on dispersion of biotissue optical properties. Laser Phys. Lett..

[18] Swartling J., Svensson J., Bengtsson D., Terike K., Andersson-Engels S. (2005). Fluorescence spectra provide information on the depth of fluorescent lesions in tissue. Appl. Opt..

[19] Miller J.P., Maji D., Lam J., Tromberg B.J., Achilefu S. (2017). Noninvasive depth estimation using tissue optical properties and a dual-wavelength fluorescent molecular probe in vivo. Biomed. Opt. Express.

[20] Khilov A., Kirillin M.Y., Loginova D., Turchin I. (2018). Estimation of chlorin-based photosensitizer penetration depth prior to photodynamic therapy procedure with dual-wavelength fluorescence imaging. Laser Phys. Lett..

[21] Khilov A.V., Kurakina D., Turchin I.V., Kirillin M.Y. (2019). Monitoring of chlorin-based photosensitiser localisation with dual-wavelength fluorescence imaging: Numerical simulations. Quantum Electron..

[22] Khilov A.V., Sergeeva E., Kurakina D., Turchin I.V., Kirillin M.Y. (2021). Analytical model of fluorescence intensity for the estimation of fluorophore localisation in biotissue with dual-wavelength fluorescence imaging. Quantum Electron..

[23] Salomatina E.V., Jiang B., Novak J., Yaroslavsky A.N. (2006). Optical properties of normal and cancerous human skin in the visible and near-infrared spectral range. J. Biomed. Opt..

[24] Kirillin M., Kurakina D., Khilov A., Orlova A., Shakhova M., Orlinskaya N., Sergeeva E. (2021). Red and blue light in antitumor photodynamic therapy with chlorin-based photosensitizers: A comparative animal study assisted by optical imaging modalities. Biomed. Opt. Express.

[25] Jacques S.L. (2010). How tissue optics affect dosimetry of photodynamic therapy. J. Biomed. Opt..

[26] Haskell R.C., Svaasand L.O., Tsay T.-T., Feng T.-C., McAdams M.S., Tromberg B.J. (1994). Boundary conditions for the diffusion equation in radiative transfer. JOSA A.

[27] Kim A.D., Ishimaru A. (1998). Optical diffusion of continuous-wave, pulsed, and density waves in scattering media and comparisons with radiative transfer. Appl. Opt..

[28] Svaasand L.O., Wyss P., Wyss M.T., Tadir Y., Tromberg B.J., Berns M.W. (1996). Dosimetry model for photodynamic therapy with topically administered photosensitizers. Lasers Surg. Med. Off. J. Am. Soc. Laser Med. Surg..

[29] Kleshnin M., Fiks I., Plekhanov V., Gamayunov S., Turchin I. (2015). Compact and fully automated system for monitoring photodynamic therapy, based on two LEDs and a single CCD. Laser Phys. Lett..

[30] Shakhova M., Loginova D., Meller A., Sapunov D., Orlinskaya N., Shakhov A., Khilov A., Kirillin M. (2018). Photodynamic therapy with chlorin-based photosensitizer at 405 nm: Numerical, morphological, and clinical study. J. Biomed. Opt..

[31] Wang L., Jacques S.L., Zheng L. (1995). MCML—Monte Carlo modeling of light transport in multi-layered tissues. Comput. Methods Programs Biomed..

[32] Svaasand L., Tromberg B., Wyss P., Wyss-Desserich M.-T., Tadir Y., Berns M. (1996). Light and drug distribution with topically administered photosensitizers. Lasers Med. Sci..

[33] Kamuhabwa A.R., Roelandts R., de Witte P.A. (1999). Skin photosensitization with topical hypericin in hairless mice. J. Photochem. Photobiol. B: Biol..

[34] Gallardo-Villagrán M., Leger D.Y., Liagre B., Therrien B. (2019). Photosensitizers used in the photodynamic therapy of rheumatoid arthritis. Int. J. Mol. Sci..

[35] Calixto G.M.F., Bernegossi J., De Freitas L.M., Fontana C.R., Chorilli M. (2016). Nanotechnology-based drug delivery systems for photodynamic therapy of cancer: A review. Molecules.

[36] Ali-Seyed M., Bhuvaneswari R., Soo K.C., Olivo M. (2011). Photolon™-photosensitization induces apoptosis via ROS-mediated cross-talk between mitochondria and lysosomes. Int. J. Oncol..

[37] Istomin Y.P., Kaplan M.A., Shliakhtsin S.V., Lapzevich T.P., Cerkovsky D.A., Marchanka L.N., Fedulov A.S., Trukhachova T.V. (2009). Immediate and long-term efficacy and safety of photodynamic therapy with Photolon (Fotolon):A seven-year clinical experience. Proc. SPIE.

[38] Trukhachova T. (2019). Safety and efficacy of photosensitizer Photolon (Fotolon) in photodynamic therapy. Proc. SPIE.

[39] Correa J., Bagnato V., Imasato H., Perussi J. (2012). Previous illumination of a water soluble chlorine photosensitizer increases its cytotoxicity. Laser Phys..

[40] Kurakina D., Khilov A., Shakhova M., Orlinskaya N., Sergeeva E., Meller A., Turchin I., Kirillin M. (2019). Comparative analysis of single- and dual-wavelength photodynamic therapy regimes with chlorin-based photosensitizers: Animal study. J. Biomed. Opt..

[41] Shliakhtsin S., Trukhachova T., Isakau H., Istomin Y. (2009). Pharmacokinetics and biodistribution of Photolon^®^(Fotolon^®^) in intact and tumor-bearing rats. Photodiagn. Photodyn. Ther..

[42] Paul S., Heng P.W.S., Chan L.W. (2013). Optimization in solvent selection for chlorin e6 in photodynamic therapy. J. Fluoresc..

[43] Isakau H., Parkhats M., Knyukshto V., Dzhagarov B., Petrov E., Petrov P. (2008). Toward understanding the high PDT efficacy of chlorin e6–polyvinylpyrrolidone formulations: Photophysical and molecular aspects of photosensitizer–polymer interaction in vitro. J. Photochem. Photobiol. B Biol..

[44] Mesradi M., Genoux A., Cuplov V., Abi-Haidar D., Jan S., Buvat I., Pain F. (2013). Experimental and analytical comparative study of optical coefficient of fresh and frozen rat tissues. J. Biomed. Opt..

[45] Wei J.C., Edwards G.A., Martin D.J., Huang H., Crichton M.L., Kendall M.A. (2017). Allometric scaling of skin thickness, elasticity, viscoelasticity to mass for micro-medical device translation: From mice, rats, rabbits, pigs to humans. Sci. Rep..

[46] Tan J., Lambie D., Sinnya S., Sahebian A., Soyer H., Prow T., Ardigò M. (2016). Histopathology and reflectance confocal microscopy features of photodamaged skin and actinic keratosis. J. Eur. Acad. Dermatol. Venereol..

[47] Heerfordt I.M., Nissen C.V., Poulsen T., Philipsen P.A., Wulf H.C. (2016). Thickness of actinic keratosis does not predict dysplasia severity or p53 expression. Sci. Rep..

[48] Fernández-Figueras M., Saenz-Sardà X., Vargas P., Thompson C., Carrato C., Puig L., Ferrándiz C., Ariza A. (2018). The depth of follicular extension in actinic keratosis correlates with the depth of invasion in squamous cell carcinoma: Implication for clinical treatment. J. Eur. Acad. Dermatol. Venereol..

[49] Sabino C.P., Deana A.M., Yoshimura T.M., da Silva D.F., França C.M., Hamblin M.R., Ribeiro M.S. (2016). The optical properties of mouse skin in the visible and near infrared spectral regions. J. Photochem. Photobiol. B Biol..

[50] Ntziachristos V., Ripoll J., Wang L.V., Weissleder R. (2005). Looking and listening to light: The evolution of whole-body photonic imaging. Nat. Biotechnol..

[51] Davis S.C., Tichauer K.M. (2016). Small-animal imaging using diffuse fluorescence tomography. In Vivo Fluorescence Imaging.

[52] Nagaya T., Nakamura Y.A., Choyke P.L., Kobayashi H. (2017). Fluorescence-guided surgery. Front. Oncol..

[53] Hurley B.R., Regillo C.D. (2009). Fluorescein angiography: General principles and interpretation. Retinal Angiography and Optical Coherence Tomography.

[54] Hamblin M.R., Huang Y. (2017). Imaging in Photodynamic Therapy.

[55] Fukuhara H., Yamamoto S., Karashima T., Inoue K. (2021). Photodynamic diagnosis and therapy for urothelial carcinoma and prostate cancer: New imaging technology and therapy. Int. J. Clin. Oncol..

[56] Leblond F., Davis S.C., Valdés P.A., Pogue B.W. (2010). Pre-clinical whole-body fluorescence imaging: Review of instruments, methods and applications. J. Photochem. Photobiol. B Biol..

[57] Kosaka N., McCann T.E., Mitsunaga M., Choyke P.L., Kobayashi H. (2010). Real-time optical imaging using quantum dot and related nanocrystals. Nanomedicine.

[58] Lavaud J., Henry M., Gayet P., Fertin A., Vollaire J., Usson Y., Coll J.-L., Josserand V. (2020). Noninvasive monitoring of liver metastasis development via combined multispectral photoacoustic imaging and fluorescence diffuse optical tomography. Int. J. Biol. Sci..

[59] Xu C.T., Svenmarker P., Liu H., Wu X., Messing M.E., Wallenberg L.R., Andersson-Engels S. (2012). High-resolution fluorescence diffuse optical tomography developed with nonlinear upconverting nanoparticles. ACS Nano.

[60] Zhu Q., Dehghani H., Tichauer K., Holt R., Vishwanath K., Leblond F., Pogue B. (2011). A three-dimensional finite element model and image reconstruction algorithm for time-domain fluorescence imaging in highly scattering media. Phys. Med. Biol..

[61] Deliolanis N.C., Dunham J., Wurdinger T., Figueiredo J.-L., Bakhos T., Ntziachristos V. (2009). In-vivo imaging of murine tumors using complete-angle projection fluorescence molecular tomography. J. Biomed. Opt..

[62] Fiks I.I., Turchin I.V. (2021). Reconstruction of fluorophore concentration distribution in diffuse fluorescence tomography based on Tikhonov regularisation and nonnegativity condition. Quantum Electron..

[63] Ong Y.H., Kim M.M., Zhu T.C. (2018). Photodynamic therapy explicit dosimetry. Photodynamic Therapy Explicit Dosimetry.

[64] Zhu W., Gao Y.-H., Song C.-H., Lu Z.-B., Namulinda T., Han Y.-P., Yan Y.-J., Wang L.-X., Chen Z.-L. (2017). Synthesis and evaluation of new 5-aminolevulinic acid derivatives as prodrugs of protoporphyrin for photodynamic therapy. Photochem. Photobiol. Sci..

[65] Chen H., Yang Z., Zou X., Wang J., Zhu J., Gu Y. (2014). Retinal injury thresholds for 532, 578, and 630 nm lasers in connection to photodynamic therapy for choroidal neovascularization. Lasers Surg. Med..

